# Tuberculosis Screening via Chest X-Ray is Financially Burdensome in Previously Independently Living Elective Total Knee Arthroplasty Patients

**DOI:** 10.51894/001c.30158

**Published:** 2022-02-24

**Authors:** Haseeb Khan, Mayank Gupta, Therese Bou-Akl, David Markel

**Affiliations:** 1 College of Human Medicine Michigan State University; 2 Ascension Providence Hospital; 3 College of Human Medicine, Michigan State University; Ascension Providence Hospital; The CORE Institute

**Keywords:** tuberculosis, chest x-ray, screening, hospital cost

## Abstract

**BACKGROUND:**

In 1995, to reduce the transmission of Tuberculosis (TB) the Centers for Disease Control and Prevention recommended that all patients discharged from hospitals be required to have chest x-rays (i.e., radiography) performed before admission to long term care facilities (LTCFs). Previously independently living patients (PILPs) who undergo elective total knee replacement (TKA) surgery are a population at higher risk to end up in LTCFs for rehabilitation. By 2017, the incidence of TB was 9,105 cases compared to 22,762 in 1995. However, the recommendations that hospitals be required to perform a chest x-ray in all patients (including PILPs) being transferred to LTCF’s have remained in place. The purposes of this study were to: a) determine the incidence of TB-positive chest x-rays in PILPS discharged to LTCFs after undergoing elective TKA surgery, and b) assess the cost (i.e., both financial and possible exposure to unnecessary radiation) of mandated chest x-rays before hospital discharge to LTCF for PILPs.

**METHODS:**

Retrospective 2012-2017 patient chart data were collected from the Michigan Arthroplasty Registry Collaborative Quality Initiative (MARCQI) to identify all elective TKAs for PILPs performed at two Ascension participating centers. Study data included sex, age, body mass index (BMI), length of stay, comorbidities, and chest x-ray results before discharge. Patients who underwent surgery for fracture, infection, trauma, or malignancy were excluded from the study. Categorical data were analyzed using Fisher’s exact test and Student’s t-test were used for continuous data.

**RESULTS:**

The authors identified 4,041 total elective TKA’s, from which 500 PILPs were discharged to a LTCF due to functional, medical and/or social concerns. Chest x-rays were associated with 500 (100%) negative findings for TB. Overall hospital costs for chest x-rays for patient’s being discharged to an extended care facility totaled $90,848.

**CONCLUSIONS:**

The mandated use of chest x-rays for TB screening of PILPs undergoing elective surgery TKA prior to discharge to LTCFs appear to place an unnecessary financial burden on the healthcare system. The mandatory use of x-rays for assessment of possible TB infection before transfer to LTCFs appears to also expose PILPs unnecessarily to radiation. Although further studies are needed to verify these results, the authors recommend that perhaps instead chest x-rays should be reserved for patients with specific comorbidities (e.g., patients on immunosuppressive therapy, with HIV, etc.) or for those patients residing in LTCFs prior to surgery.

## INTRODUCTION

Tuberculosis (TB) is an infectious disease caused by *Mycobacterium tuberculosis* bacteria. The disease usually affects the lungs, but TB bacteria can attack any part of the body.^1,2^ The disease spreads between persons through air contaminated with particulate dissemination through coughs, sneezes, or saliva.^1,2^ Through this route anybody can contract the bacterium, although not everyone becomes sick.^2^ Fortunately, TB is a preventable and treatable disease, though one that can be fatal if left untreated.^2^ Screening and treatment for TB is particularly important among higher-risk persons to mitigate the risk of TB transmission to those living in close contact with them.^1,2^

Adults greater than 65 years of age (referred to as “elderly” in this paper) have the highest case rate for TB infection in the United States.^1,2^ Compared to other residential units, elderly patients make up more than half of long-term care facility (LTCF) residents. Examples of LTCFs include adult foster care homes, board and care homes and any other facility that allows for the congregation of elderly persons. In 1987, 6,150 TB cases were reported for elderly people. This accounted for 27% of the total U.S. TB morbidity while only representing 12% of the population.^1^ This increased risk of contracting TB is likely due to the communal nature of these facilities.^3,4^ To reduce the transmission of TB, in 1995 the CDC recommended that all patients should have a screening chest x-ray (i.e., radiograph) before discharge from a hospital to LTCFs.^2^ Although chest x-ray is an important diagnostic method, it is also associated with radiation exposure and increased financial burden on both the hospital and patients.^5,6^

Total knee arthroplasty (TKA) is an effective definitive treatment in patients who fail nonoperative treatments of end stage osteoarthritis and typically results in a decrease of osteoarthritic pain, improved mobility, and better quality of life.^7–10^ It is one of the most performed orthopaedic procedures, with over 500,000 TKA’s annually performed in the US.^9^ Further, the annual demand for a first knee joint replacement procedure also called primary TKA is projected to grow by 673% to 3.48 million procedures by 2030.^11^

After TKA surgery, some previously independently living patients (PILPs) require placement at LTCFs rather than being discharged directly home due to ongoing or postoperative functional, social, and/or safety concerns. If a PILP is to be discharged to an LTCF it is currently required that he/she undergo a chest x-ray study to rule out the presence of TB before they are transferred.^2^

### Purposes of Study

The purposes of this study were to: a) determine the incidence of TB-positive chest x-rays in PILPs discharged to a LTCF after undergoing TKA surgery, and b) assess the cost (i.e., financial, and possible exposure to unnecessary radiation) of mandated chest x-rays before hospital discharge to LTCFs for PILPs.

## METHODS

Approval by the authors’ Ascension Providence Hospital Institutional Review Board was received to access patient medical chart data from Ascension Providence Southfield and Ascension Providence Novi hospital’s Michigan Arthroplasty Registry Collaborative Quality Initiative (MARCQI) database. The MARCQI is a database of over 370,000 total joint arthroplasty procedures. The database includes information pertaining to patient demographics, diagnoses, clinical indications, surgical techniques, and type of TKA.

The MARCQI database was queried for all TKA patients irrespective of their age over a five-year period from March 2012 to March 2017 (total 4,041). Of this initial sample, 500 (12.4%) PILPs were discharged to a LTCF and their cases reviewed. Prior to surgery, these patients used to live independently and took care of themselves. After surgery they were discharged to LTCFs to complete the postoperative physiotherapy and regain their locomotive capabilities. Patients who received a TKA because of fracture or infection and trauma, or malignancy were excluded due to the potential for decreased immunity in these patients.

Patient demographic data, as well as data on medical comorbidities, length of stay, complications, and discharge disposition were also collected. These data were specifically abstracted by two nurse clinical data abstractors from the Ascension hospitals’ quality departments. The financial data were provided by the Ascension Providence finance team.

Net procedure costs were determined by reviewing the gross charges, charge codes for chest x-rays (either single and/or two view) and the Medicare fee schedule and charge codes for radiologist fees. The net revenue was calculated based on 75% reduction of the gross charges. Next, net profits were calculated by subtracting the x-ray fees and the radiologist fees from net revenues.

### Statistical Analysis

Frequency and percentage data were calculated. Statistical analysis was performed by first author HK using Microsoft Excel (Microsoft Office 365, Version 1705) software.

## RESULTS

### Patient Characteristics

From March 2012 through March 2017, a total of 4,041 TKAs were completed at the two selected Ascension Providence hospital sites. Of these, 500 (12.4%) PILPs underwent unilateral TKA and were discharged to an LTCF after having a screening chest x-ray. This patient population consisted of 98 (19.64%) men and 402 (80.4%) women. Average patient age was 76.6 (SD = 7.0) and ranged from 65 through 95 years with a mean BMI of 32.14 (SD = 6.98). Average length of hospital stay was 3.35 days (SD = 2.62 days). Other demographic and clinic information such as self-reported pre-operative history of bleeding disorder, 8/441 (1.8%), deep vein thrombosis, 45/441 (9.0%), and diabetes, 73/347 (14.6%) were also considered. (Table 1).

**Table 1.** Summary of Patient demographic data, medical comorbidities, length of stay, complications, and discharge disposition

**Gender**	
Male	98 (19.6%)
Female	402 (80.4%)
Total	500
**Age (in complete years)**	
Mean	76.6 (SD = 7)
Median	76
Range	65 - 95
**Length of Stay (days)**	
Mean	3.3 (SD = 2.62)
Median	3
Range	1 - 56
**BMI (units)**	
Mean	32.14 (SD = 6.98)
Median	31.46
Range	17.85 - 61.2
Pre-Op bleeding disorder*	8/441 (1.8%)
Pre-op History of DVTs*	45/441 (10.0%)
Pre-op Diabetes*	73/347 (21%)

BMI = body mass index

DVT = deep vein thrombosis

*self-reported

### Financial Analyses

Of 500, 15 (3%) patients received a two-view x-ray, while the remaining 485 (97.0%) received a single-view chest x-ray prior to discharge to a LTCF. For two-view chest x-ray, the charge (i.e., Gross Revenue) was $210.09, Net Revenue (after 75% contractual write-off) $52.52, Cost per chest x-ray: $28.79, Radiologist professional fee: $11.17 and the net profit was $12.56. This financial data is summarized in Table 2.

**Table 2.** Summary of financial data

	**Gross revenue**	**Net revenue**	**Cost per chest x-ray**	**Radiologist fee**	**Net profit**
**Two-view chest x-ray**	$210.09	$52.52	$28.79	$11.17	$12.56
**Single-view chest x-ray**	$179.34	$44.64	$24.47	$9.37	$10.80

For single-view chest x-rays, the Gross Revenue Charge was $179.34, Net Revenue (after 75% contractual write-off) $44.64, Cost per chest x-ray $24.47, and Radiologist professional fee $9.37 and the net profit is calculated to be $10.80. (Table 2)

### Radiograph Results

The use of single-view (n = 485 patients) and two-view (n = 15 patients) chest x-ray correlated to a total of 500 negative findings for TB. Other findings not related to the study and incidentally found on chest x-ray were Cardiomegaly (i.e., abnormal enlargement of the heart), 70 (14%), Hiatal Hernia, 5 (1%), Granuloma (i.e., a small area of inflammation typically appearing in the lungs), 4 (0.8%), and Pulmonary Fibrosis (i.e., lung disease consisting of damaged or scarred lung tissue), 1 (0.2%).

Overall, the use of chest x-ray was not associated with detection of TB in any of the 500 sample patients. In addition, the overall hospital and physician costs for chest x-rays totaled $90,848.

## DISCUSSION

In 2017, a total of 9,105 TB cases (i.e., a rate of 2.8 cases per 100,000 persons) were reported in the US. This was a decrease from 9,252 (at a rate of 2.9 per 100,000 persons) the number of cases reported in 2016 and the lowest case count on record in the US since TB reporting began in 1953.^12^ However, as of the writing of this manuscript, the CDC still recommends that all patients discharged from a hospital to LTCFs have a chest x-ray before admission to a LTCF.^2^ Our study findings suggest that for those hospital TKA PILPs being transferred to a LTCF post-surgery, receiving chest x-ray for TB screening prior to discharge to a LTCF may not only place an unnecessary financial burden on health systems, but may also be exposing patients to radiation unnecessarily as well.

Currently, the highest TB incidence rates in the U.S. occur among 45 - 65-year-old and patients > 65 years old.^13^ Our study patients had a mean population age of 77 (SD = 7.0) years and consisted of patients who were all PILPs and electively chose to undergo TKA. The authors did not find a single positive radiograph in the highest risk age group for TB in this study population.

Chest x-rays could instead be reserved as a screening tool for higher risk patients, patients with specific comorbidities, or elderly individuals already residing (i.e., prior to surgery) in LTCFs. Patients who have had close contacts of people suspected to have TB or those who have had recent travel to countries with higher TB incidence rates are also at higher risk due to the highly contagious nature of the disease.^14^ Co-morbidities that increase the risk of contracting TB are those that impair the immune response such as diabetes, malnutrition, and HIV.^15^ Patients on immunosuppressive therapy (e.g., transplant patients and rheumatoid patients on TNF-alpha inhibitors) are also at increased risk of contracting TB.^16^

Multiple studies have demonstrated that diagnosing TB can be difficult in elderly patients when depending solely on radiologic findings.^17,18^ Studies have shown radiographs with decreased findings of pulmonary cavitation and nodules on chest x-ray in elderly patients who were diagnosed with TB.^17–19^ There is also no standardized method of reporting chest x-ray results in TB cases. This is due to the variations in radiological finding assessments, which could lead to increased inter-observer variability.^20,21^ Studies that compared the sensitivity and specificity of TB screening via chest x-ray found a sensitivity of 73–79% and a specificity of 60–63% in a high-risk population.^20^ This implies that, theoretically only 73 – 79% of patients who truly had TB would be identified by chest X-rays alone.

### Study Limitations

First, chest x-ray findings are based entirely on individual radiologist interpretation, i.e., are subjective and not evaluated using standardized criteria. Several standardized approaches to report chest x-rays have been proposed but are not yet universally accepted.^21^ Second, our PILPs, i.e., general population elective TKA surgery patient sample may not have been exposed to TB and thus may not provide an accurate representation of exposure to TB in the general elderly population.

In addition, the authors were unable to determine the purified protein derivative (PPD) status to measure sample patients’ presence or absence of immunity against TB. For future studies, this information could be relevant in examining possible risk factors and potential confounders for TB transmission or recurrence.^22–24^ Confirming prior TB exposure of elderly patients is also important since the disease can be latent for several years before patients may manifest any symptoms.^24,25^

## CONCLUSIONS

During our study, we were not able to detect a single case of TB in PILPs when following current CDC recommendations. The authors conclude that the 1995 CDC recommendation for chest x-ray as a discharge and admission requirement for hospital to LTCF for all patients may need to be revised to decrease unnecessary medical tests, radiation exposure, and overall healthcare system costs among those PILPs being transferred to LTCFs after undergoing elective TKA surgery.

In addition, the authors also recommend the CDC criteria should be revised to systematically consider an TKA patient’s pre-operative host factors and environmental factors when deciding to screen for TB. Additional studies investigating more cost-effective and patient-specific TB screening methods in TKA surgery PILPs being discharged to LTCF are needed.

### Conflict of Interest

Support for the Michigan Arthroplasty Registry Collaborative Quality Initiative is provided by Blue Cross and Blue Shield of Michigan (BCBSM) and Blue Care Network as part of the BCBSM Value Partnerships program. The opinions, beliefs and viewpoints expressed by the author do not necessarily reflect the opinions, beliefs, and viewpoints of BCBSM or any of its employees.
